# Supplementary material for: 5-Hydroxymethylcytosine localizes to enhancer elements and is associated with survival in glioblastoma patients
Source: Nat Commun. 2016 Nov 25;7:13177. doi: 10.1038/ncomms13177 (PMC5133638; doi:10.1038/ncomms13177)
Supplement: Supplementary Information — Supplementary Figures 1-8 and Supplementary Tables 1 & 3 [file ncomms13177-s1.pdf]

SUPPLEMENTARY FIGURES:

Supplementary Figure 1

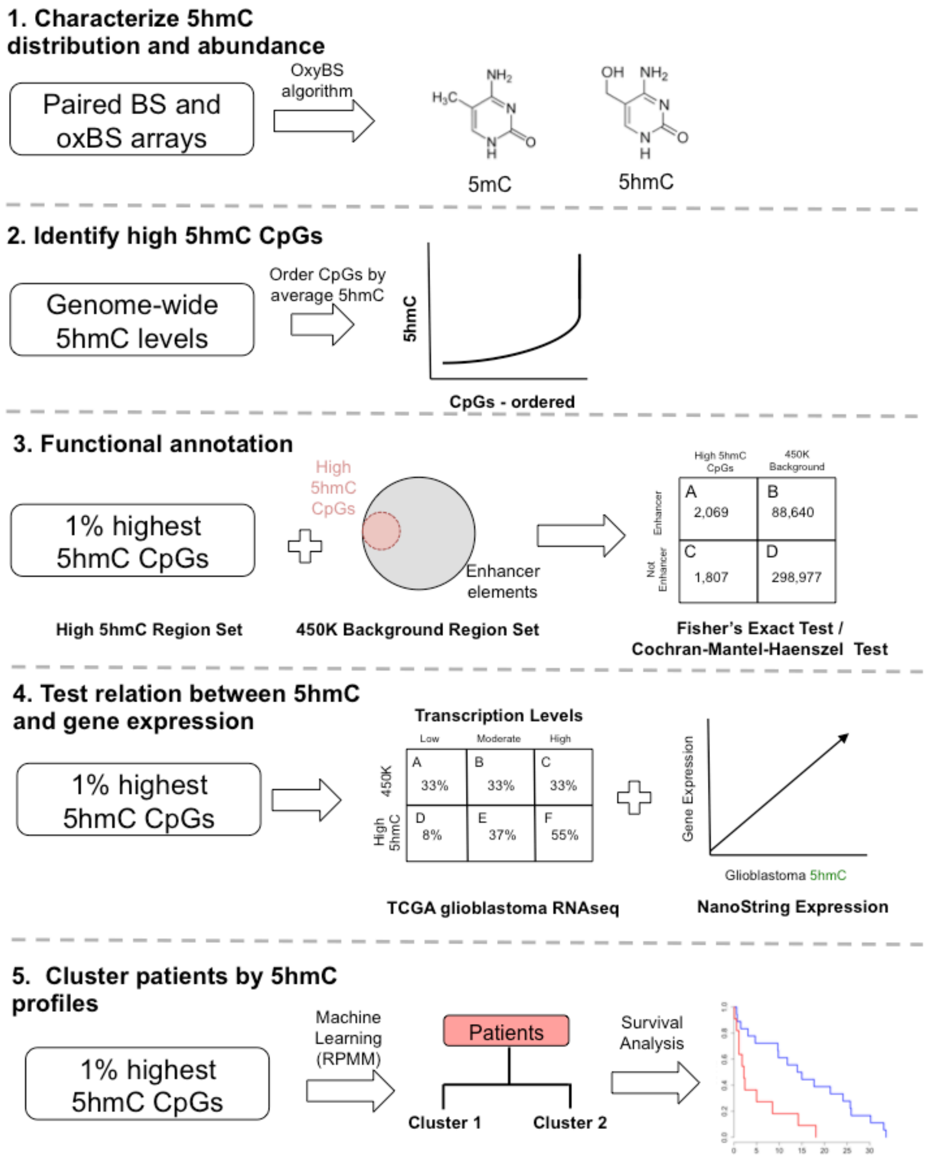

Supplementary Figure 1. Glioblastoma 5hmC quantified by paired BS and oxBS treated DNA hybridized to Infinium DNA methylation arrays. Workflow depicts analytic approach using the BS-oxBS protocol and OxyBS software. 1. Nucleotide level 5mC and 5hmC are resolved from paired arrays. 2. The CpGs in the glioblastoma genome that exhibit frequent levels of high 5hmC are identified. 3. The enrichment of genomic regions with high 5hmC is tested against the background of the 450K array genomic regions. 4. Correlations between 5hmC and glioblastoma are assessed in glioblastoma TCGA RNAseq data and confirmed using NanoString nCounter candidate genes. 5. The association between patient 5hmC profiles and survival are analyzed.

Supplementary Figure 2

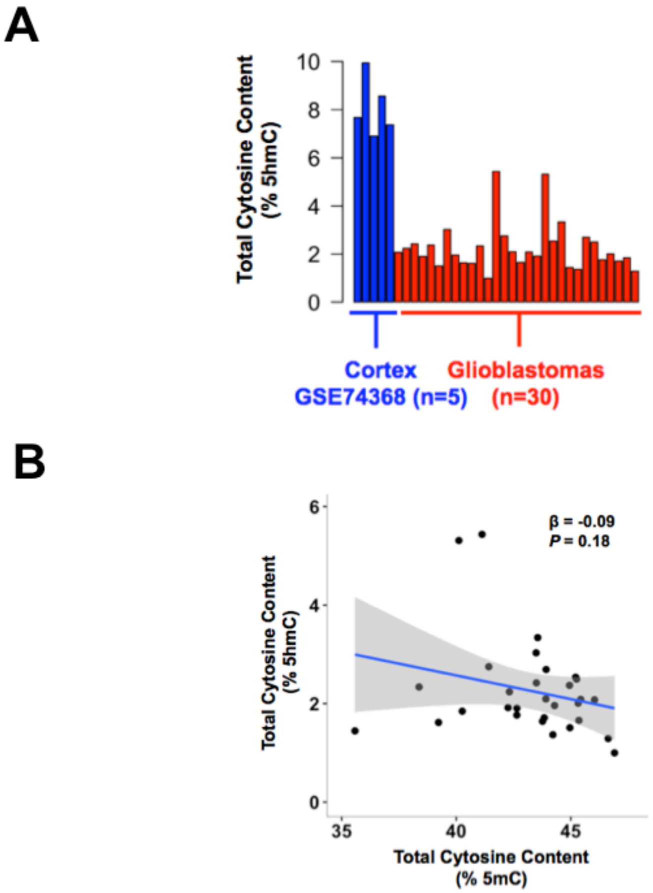

Supplementary Figure 2. (A) Proportion of 5hmC in total cytosine content (a measure of total 5hmC) across prefrontal cortex samples from GSE74368 (n=5) is elevated compared with glioblastomas (n = 30). **(B)** Proportion of 5hmC in total cytosine content is not significantly associated with the proportion of 5mC in total cytosine content in glioblastoma (n=30).

Supplementary Figure 3

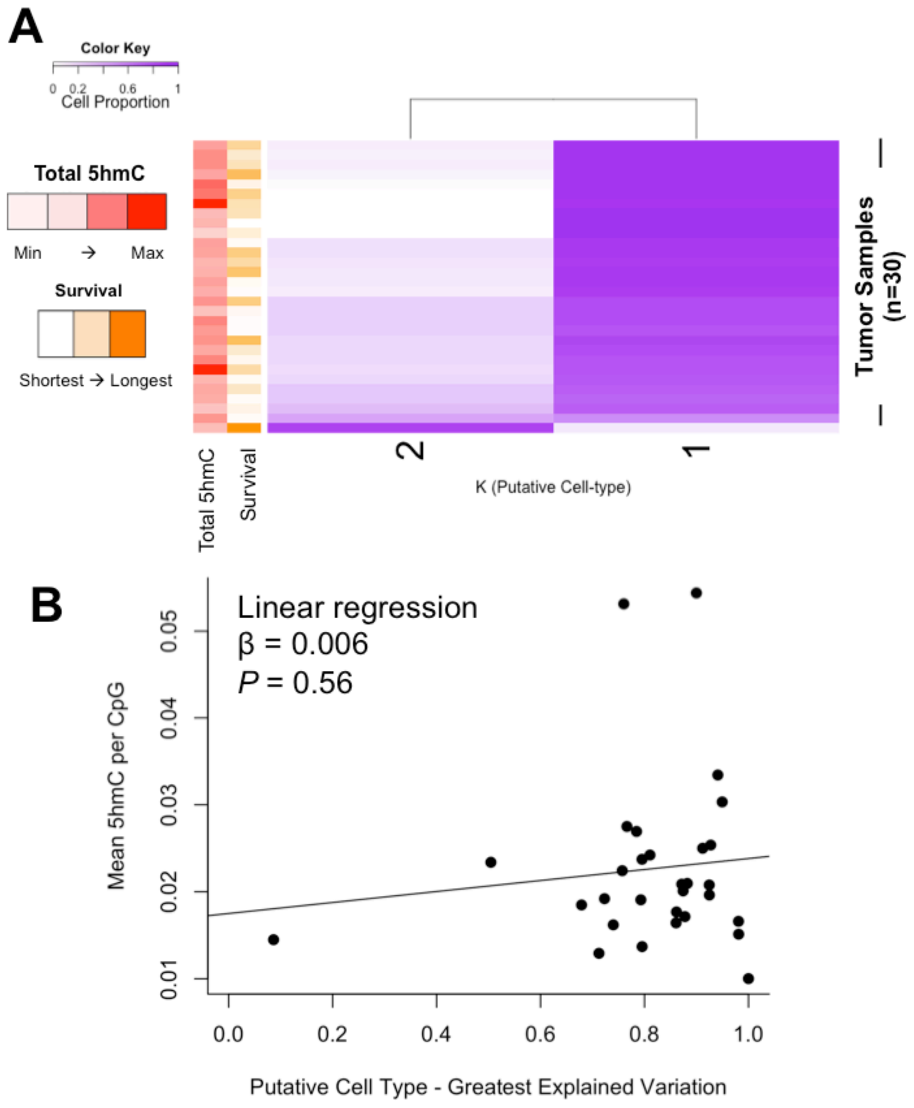

Supplementary Figure 3. (A) Clustering heatmaps of cell proportion matrix for glioblastoma data set (purple intensity indicates cell proportion). **(B)** Putative cell-type 1 (source of greatest explained variation) was not significantly associated with proportion of 5hmC in total cytosine content ($P > 0.05$, linear regression).

Supplementary Figure 4

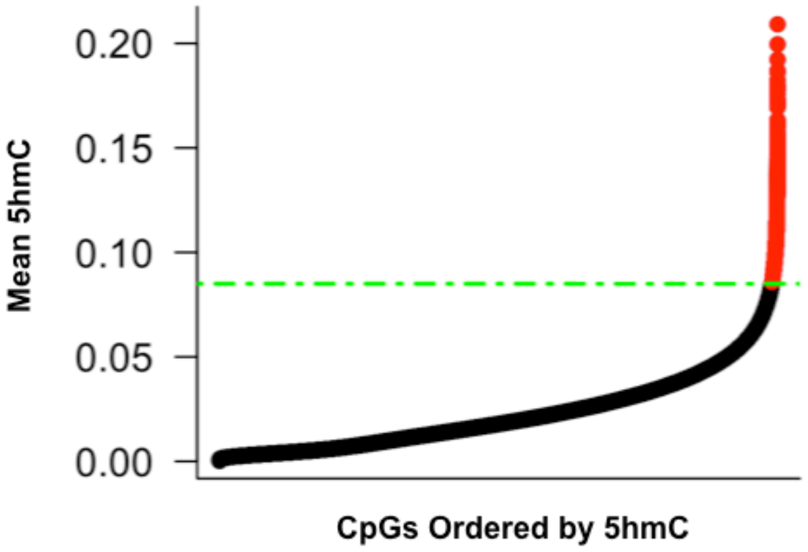

Supplementary Figure 4. CpG-specific mean 5hmC values were calculated across the 450K array and ordered by increasing magnitude. The CpGs with the highest one-percent 5hmC values were termed high 5hmC CpGs (red points).

Supplementary Figure 5A

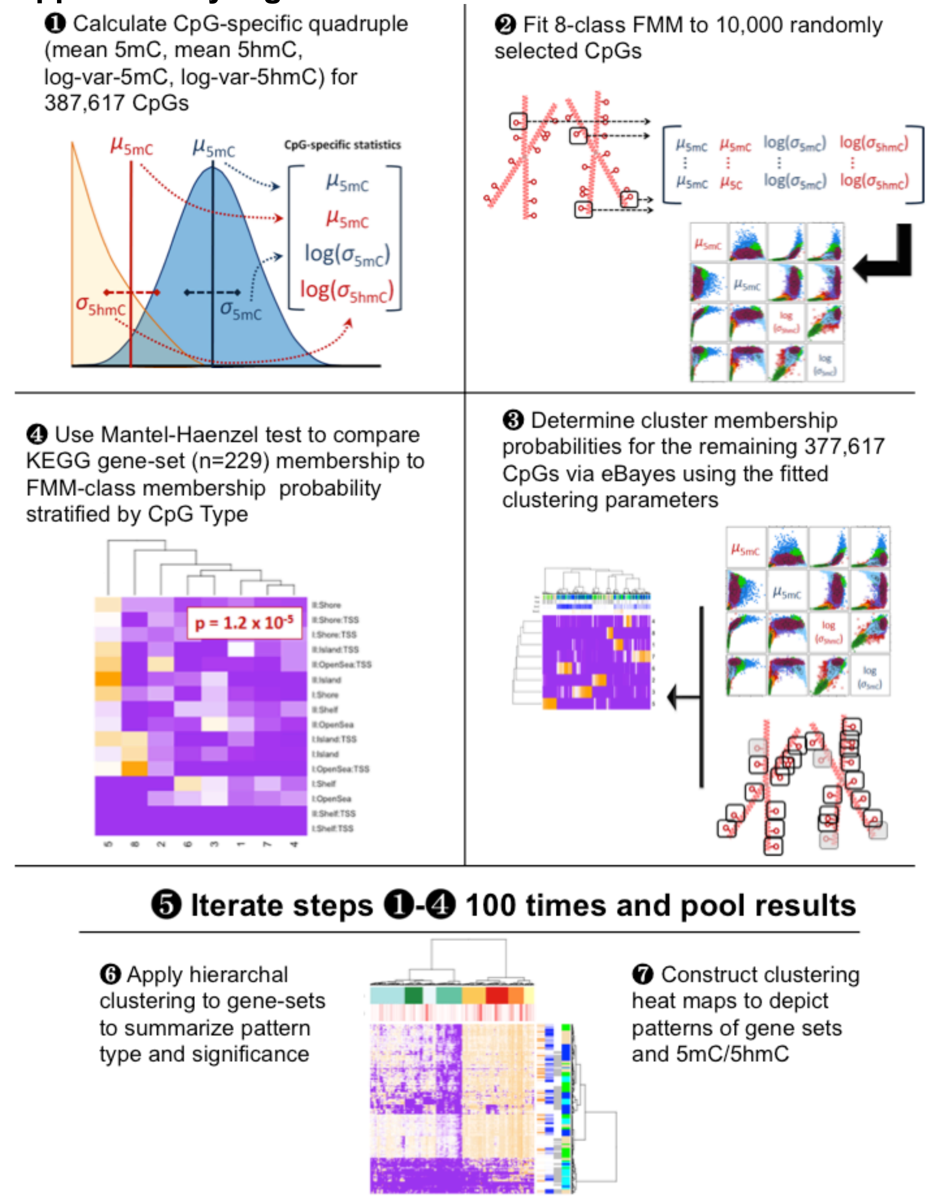

Supplementary Figure 5B

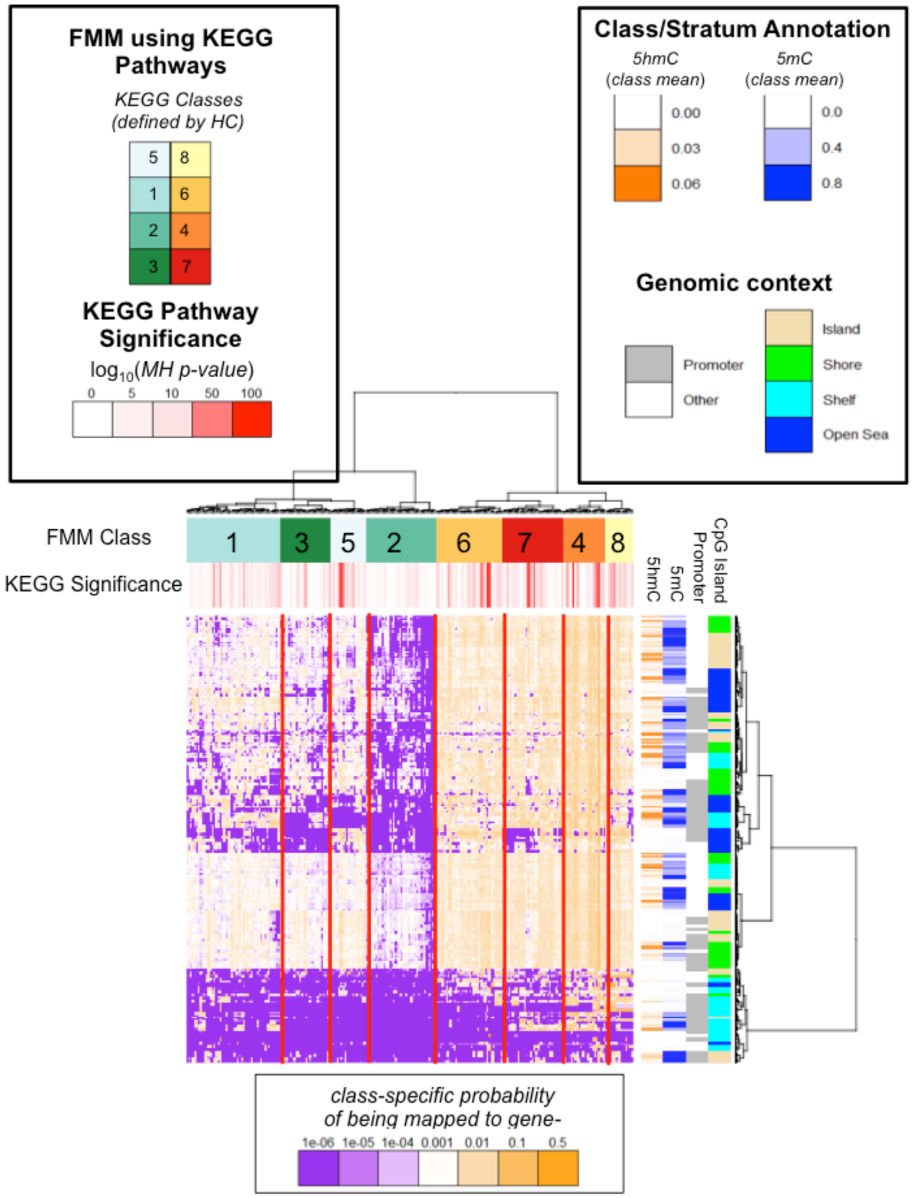

Supplementary Figure 5C

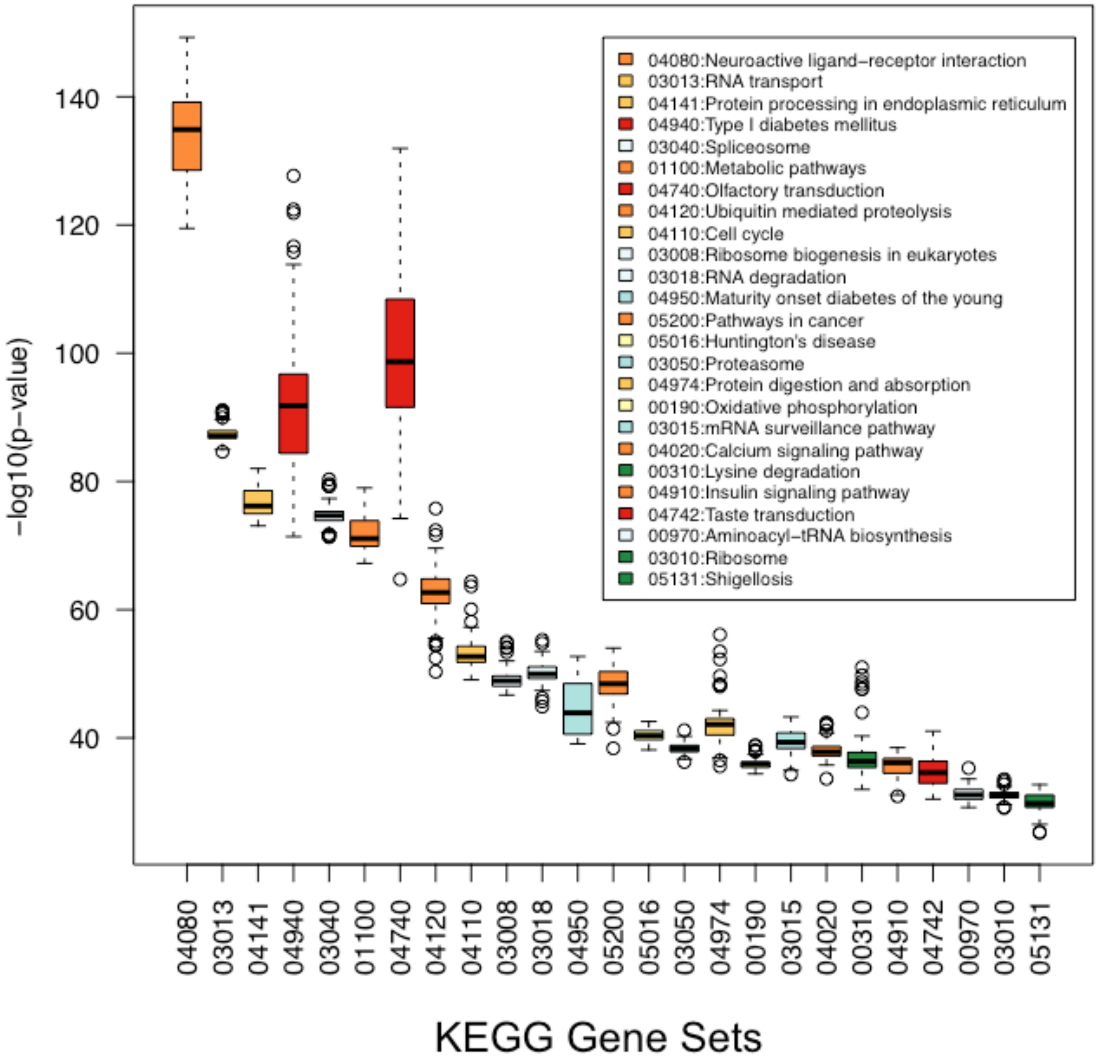

Supplementary Figure 5. (A) Analysis strategy for agnostic consensus clustering approach. **(B)** Consensus clustering using KEGG gene-sets to determine functional relevance of 5hmC and 5mC patterns in glioblastoma. **(C)** Boxplots of top-associated gene-sets.

Supplementary Figure 6

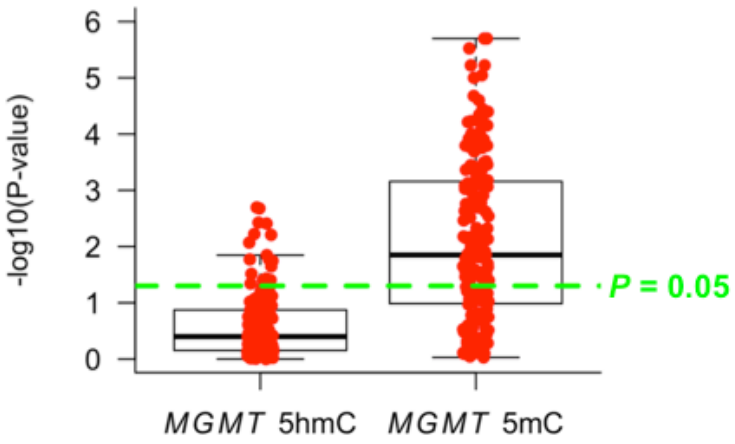

Supplementary Figure 6. Comparison of significance tests (Spearman's rho test) for CpG-specific 5hmC and 5mC values and *MGMT* gene expression (n=176 CpGs).

Supplementary Figure 7

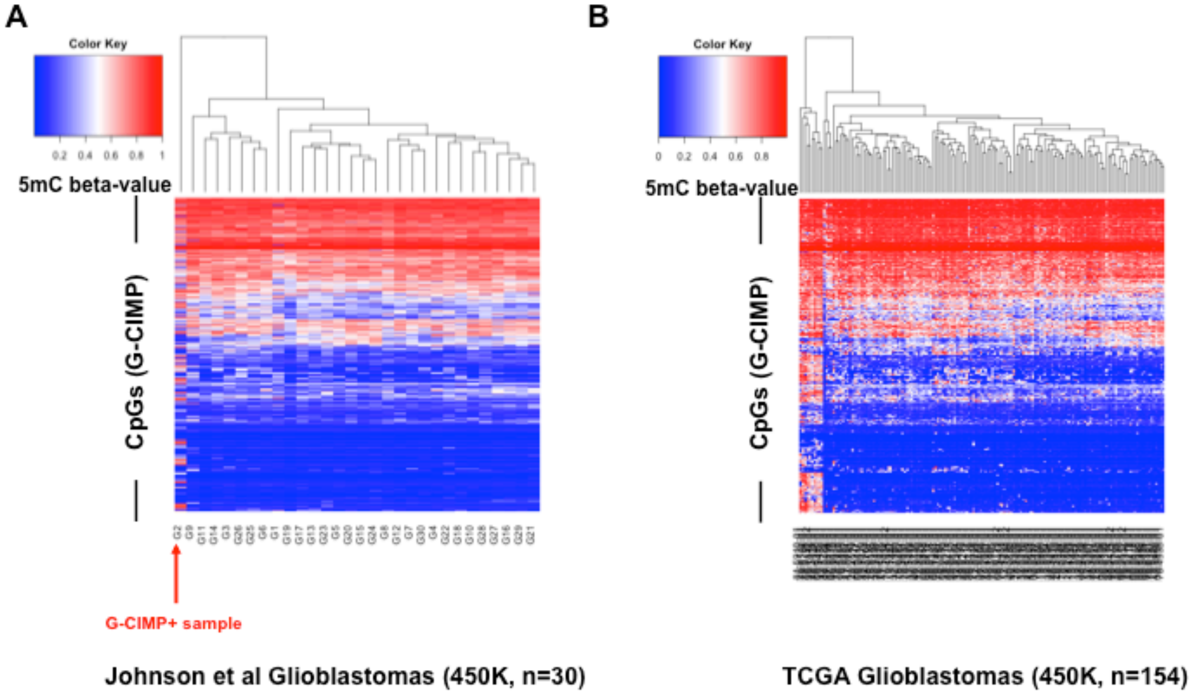

Supplementary Figure 7. Identification of non-IDH-mutant Glioma-CpG Island Methylator Phenotype (G-CIMP) tumor sample in glioblastoma cohort (n=30). **(A)** Heat map of 5-methylcytosine values and unsupervised hierarchal clustering of CpGs from G-CIMP genes identified in Noushmehr et al [8]. High levels of methylation, that is outlier DNA methylation, at these genes is suggestive of a G-CIMP phenotype for the sample in the furthest left portion of the dendrogram **(B)** Unsupervised hierarchal clustering of CpGs from G-CIMP genes in the TCGA glioblastoma data set (n=154). High levels of DNA methylation at G-CIMP genes are present in the far left branch of the dendrogram.

Supplementary Figure 8

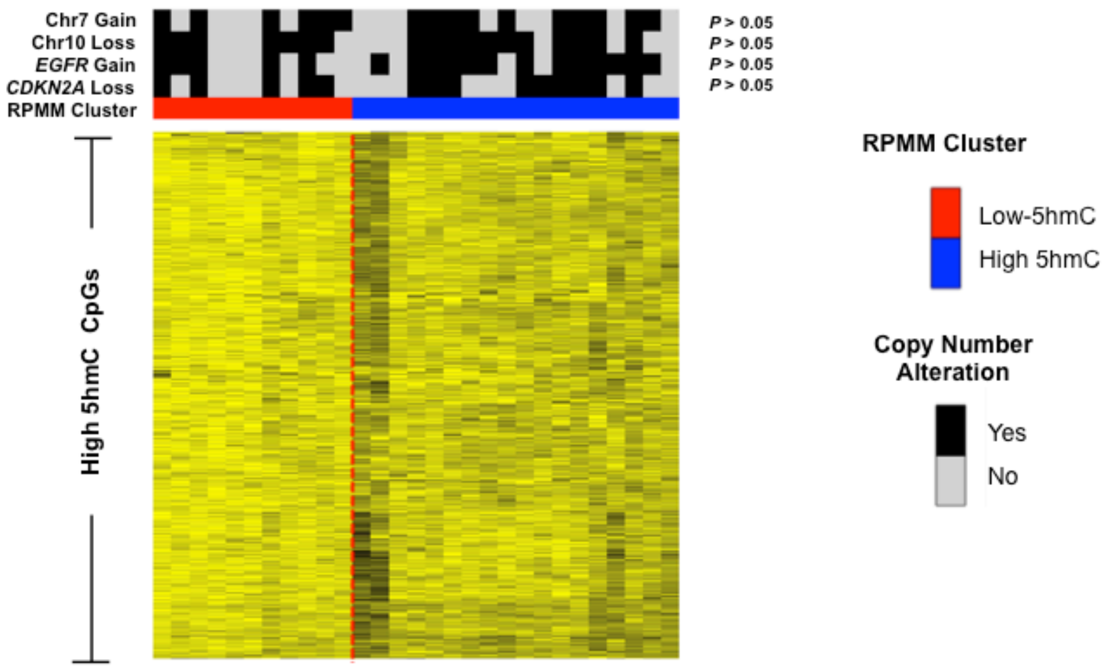

Supplementary Figure 8. Recursively Partitioned Mixture Model (RPMM) of tumor samples based on highest 5hmC CpGs (n=3,876 CpGs). In the heat map, each row represents a single CpG and each column represents a single tumor sample. Present or absence of copy number alterations for chromosome 7 gain, chromosome 10 loss, *EGFR* gain, and *CDKN2A* loss were not associated with cluster membership ($P > 0.05$, Fisher's exact test).

SUPPLEMENTARY TABLES:

Supplementary Table 1. Correlations between 5hmC levels and gene expression of epigenetic enzymes

Gene	Spearman Correlation Coefficient	Spearman P-value
DNMT1	-0.106	0.630
DNMT3A	0.272	0.209
DNMT3B	-0.190	0.385
IDH1	-0.302	0.161
IDH2	0.019	0.933
TET1	0.158	0.469
TET2	-0.069	0.754
TET3	0.151	0.489

Supplementary Table 2. Genes actively transcribed and with alternative mRNA splicing are characterized by 5-hydroxymethylcytosine in glioblastoma.

Gene transcription levels (RNAseq, n=172 TCGA glioblastoma)

	**Gene transcription levels (TCGA, n=172)**			
	**Low**	**Moderate**	**High**	***P*-value**
All Genes on 450K array (n=20,621 genes)	6,397 (32.6 %)	6,582 (33.6 %)	6,621 (33.8 %)	
Genes with 5hmC sites (n=2,121 genes)	174 (8.1 %)	785 (36.7 %)	1,183 (55.2 %)	5.20E-139

*Pearson's Chi_Squared test

Supplementary Table 3

TCGA glioblastoma-specific splicing events (RNAseq, n=160 TCGA glioblastoma)

Alternative Transcription Events	**All Genes on 450K* array (n=20,621 genes)**	**Genes with 5hmC sites (n=2,121 genes)**	**Enrichment (95 % CI)**	***P*-value**
Exon Skip (ES)	5,008 (24.3 %)	837 (39.5 %)	2.03 (1.85 - 2.23)	2.23E-48
Alternate Donor Site (AD)	1,569 (7.6 %)	179 (8.4 %)	1.12 (0.95 - 1.31)	1.70E-01
Alternate Acceptor Site (AA)	1,821 (8.8 %)	269 (12.7 %)	1.50 (1.30 - 1.72)	2.36E-08
Retained Intron (RI)	1,258 (6.1 %)	123 (5.8 %)	0.95 (0.78 - 1.15)	6.30E-01
Mutually Exclusive Exons (ME)	96 (0.4 %)	22 (1.0 %)	2.24 (1.33 - 3.60)	2.00E-03
Alternate Terminator (AT)	2,327 (11.1%)	353 (16.6 %)	1.57 (1.38 - 1.78)	3.44E-12
Alternate Promoter (AP)	2,580 (12.5 %)	483 (22.8 %)	2.06 (1.84 - 2.30)	9.75E-35

* Genes on the 450K array with CpGs that passed QC (n=387,617). The level of enrichment was determined by Fisher's exact test by testing binary comparisons (e.g., gene region of interest vs. others)
